# Early warning for human mental sub-health based on fMRI data analysis: an example from a seafarers' resting-data study

**DOI:** 10.3389/fpsyg.2015.01030

**Published:** 2015-07-23

**Authors:** Yingchao Shi, Weiming Zeng, Nizhuan Wang, Shujiang Wang, Zhijian Huang

**Affiliations:** Lab of Digital Image and Intelligent Computation, Shanghai Maritime UniversityShanghai, China

**Keywords:** fMRI, default mode network, support vector machine, mental, seafarer

## Abstract

Effective mental sub-health early warning mechanism is of great significance in the protection of individual mental health. The traditional mental health assessment method is mainly based on questionnaire surveys, which may have some uncertainties. In this study, based on the relationship between the default mode network (DMN) and the mental health status, we proposed a human mental sub-health early warning method by utilizing two-fold support vector machine (SVM) model, where seafarers' fMRI data analysis was utilized as an example. The method firstly constructed a structural-functional DMN template by combining the anatomical automatic labeling template with the functional DMN extracted by independent component analysis. Then, it put forward a two-fold SVM-based classifier, with one-class SVM utilized for the training of the initial classifier and two-class SVM utilized to refine the classification performance, to identify seafarers' mental health status by utilizing the correlation coefficients (CCs) among the areas of structural-functional DMN as the features. The experimental results showed that the proposed model could discriminate the seafarers with DMN function alteration from the healthy control (HC) effectively, and further the results demonstrated that when compared with the HC group, the brain functional disorders of the mental sub-healthy seafarers mainly manifested as follows: the functional connectivity of DMN had obvious alteration; the CCs among the different DMN regions were significant lower; the regional homogeneity decreased in parts of the prefrontal cortex and increased in multi-regions of the parietal, temporal and occipital cortices; the fractional amplitude of low-frequency fluctuation decreased in parts of the prefrontal cortex and increased in parts of the parietal cortex. All of the results showed that fMRI-based analysis of brain functional activities could be effectively used to distinguish the mental health and sub-health status.

## Introduction

Under the strain of modern life, many people are in a certain sub-health state between health and disease. The early detection and careful nursing can make the sub-healthy people recuperate to their health; on the contrary, the sub-health status is likely to further develop into disease. Mental sub-health is one of series of sub-health status, and mainly performed as unexplained mental fatigue, mood disorders, thought disorders, panic, anxiety, low self-esteem, nervous, reckless, even suicidal thoughts. Mild mental sub-health status may not influence individuals' life or work temporarily, but for some special occupations such as seafarers, their mental health status seriously affects the efficiency and the success or failure of their jobs. The poor mental or psychological state even may be prone to accidents, which could do damage to the patients and their families, even to the society. For example, for the seafarers, because of the loneliness, separation from families, stress, lack of shore leave, short ship turn abound times, job security and cultural problems, etc. (Iversen, 2010), working at sea certainly was potential to be fatiguing, and it might be linked to longer-term individual ill-health, such as anxiety and depression, etc., which may leave serious hazards for the safe maritime operations (Wadsworth et al., 2008). An accepted statistical result from the international maritime community showed that 80% of the marine casualties were caused by human factors. Thus, the prediction and discovery of the mental disorders are very important, promising and challenging, especially in the early stage. However, as far as we know, there are still little quantitative and objective methods for mental health evaluation. The traditional mental health assessment methods were mainly based on questionnaire surveys, which might not assess the mental health status accurately due to the incomplete design of questionnaire or subjective wills of testees.

Neuropsychology is a special branch of psychology that focuses on brain functioning, which is used to explore the structure and function of the brain as they relate to specific psychological processes and behaviors. It was advocated that the mental is the brain's subjective response for the objective reality, and the brain is the operating platform of the mental (Giles, 2002). Thus, abnormal brain functional activities likely reveal some psychiatric disorders, which probably affect the higher-level cognitive psychological activity, such as introspection, memory, consciousness, sensation and social interaction, etc.

Human brain is the most complex organization, and it controls our emotions, memories, dreams, hopes, fears and all subconscious or conscious minds. Among various of brain research methods, functional magnetic resonance imaging (fMRI) is a kind of powerful, safe and non-invasive method for the study of human brain function with high spatial resolution and relatively good temporal resolution (Poldrack et al., 2011), and is extensively used by the psychologists, psychiatrists and neurologists to analyze the mental function of human individuals. FMRI technology was originally applied to studying brain activation under the designed tasks; however, it has limitations when participants were unable to better execute the pre-designed task. Fortunately, resting-state fMRI modality has provided a new opportunity for the study of brain's intrinsic activities.

In 1995, Biswal and his colleagues firstly showed the spontaneous low frequency fluctuations (<0.08 Hz) in the blood oxygen level dependent (BOLD) fMRI signal, and these spontaneous fluctuations were coherent within some specific neuro-anatomical systems such as the somatomotor system (Biswal et al., 1995). This phenomenon can be called functional connectivity (FC) (Friston, 1994), which means the correlations of physiological signals recorded from spatially distinct brain regions. Some other studies reported that this coherent phenomenon also existed in several other brain systems, such as visual, auditory, and language processing cortices (Lowe et al., 1998, 2000). Furthermore, Raichle and his colleagues firstly proposed the DMN, which provided a new perspective for the resting-state fMRI study, and was widely used in the studies of cognitive neuroscience, neuropsychology and clinical medicine, etc. (Orrù et al., 2012). Several reports have informed that DMN played important roles in the higher cognitive activities of human beings, such as monitoring the external environment and supporting internal mentation (Ghatan et al., 1995; Raichle et al., 2001; Kelley et al., 2002; Mitchell et al., 2006; Gilbert et al., 2007; Gruber et al., 2009). The previous studies reported that the DMN mainly consisted of several regions including the medial prefrontal cortex (MPFC), posterior cingulated cortex (PCC) or precuneus, and bilateral inferior parietal cortices (bIPC), which could be located in the prefrontal, parietal, temporal and occipital cortices. Among these brain areas, prefrontal cortex acted a great part in planning complex cognitive behavior (Yang and Raine, 2009), such as the personality expression, decision making and moderating social behavior, etc.; the parietal cortex integrated sensory information from different modalities, particularly determining spatial sense and navigation (Penfield and Rasmussen, 1950); the temporal cortex was involved in the retention of visual memories, processing sensory input, comprehending language, storing new memories, emotion and deriving meaning (Squire and Zola-Morgan, 1991); and the occipital cortex was the visual processing center of the brain which also had the function related to memory and motion perception (Sveinbjornsdottir and Duncan, 1993).

Numerous studies using the resting-state fMRI modality demonstrated that contrast to the healthy control (HC) group, patients with neurological or psychiatric disorders suffered the abnormality of spontaneous neural activity in certain brain areas, especially in DMN. For example, Greicius et al. (2004) reported that the Alzheimer's disease group showed decreased resting-state activity in the posterior cingulated cortex and hippocampus, suggesting that disrupted connectivity between these two regions may account for the posterior cingulated hypometabolism commonly detected in positron emission tomography studies of early Alzheimer's disease. Bluhm et al. (2007) also reported that schizophrenic patients had significantly less correlation between spontaneous slow activity in the posterior cingulated and that in the lateral parietal, medial prefrontal, and cerebellar regions. Connectivity of the posterior cingulated cortex was found to vary with both positive and negative symptoms in schizophrenic patients (Bluhm et al., 2007). Besides, recently, regional homogeneity (ReHo) (Zang et al., 2004) and fractional amplitude of low-frequency fluctuation (fALFF) (Zou et al., 2008) were also used to explore the alteration of DMN activity. For example, the schizophrenia patients were reported with a decrease of ReHo, which distributed over the bilateral frontal, temporal, occipital, cerebellar posterior, right parietal and left limbic lobes (Liu et al., 2006), and researchers also found significant abnormal ReHo in resting brain in first-degree relatives of schizophrenic patients (Liao et al., 2012); the ADHD (Attention-deficit hyperactivity disorder) patients showed a significant increased fALFF in the bilateral lingual gyrus, right precentral gyrus and left cuneus and a decrease in the cerebellum, the bilateral superior frontal gyrus and middle frontal gryus (Cheng et al., 2012).

However, currently, most of the resting-state fMRI-based researches mainly focus on investigating the brain functional disorders of the mental-illness or psychiatric patients in comparison with the healthy controls. There are still few studies paying attention to the active detection of mental disorders. Resting-state fMRI-based DMN functional connectivity analysis is significant for the exploration of brain's neural physiological activities in clinical applications. The abnormalities of DMN may contribute to a variety of symptomatic behaviors in diseases. Here we regarded the abnormal FC of DMN as the criterion of mental sub-health, and the FC was measured by using the Pearson correlation coefficients (CCs) among different DMN regions. Thus, precise positing method for ROIs was a key for guaranteeing the reliability of CCs, and we firstly established the structural-functional DMN by intersecting the structural and functional masks, which could remedy some shortcomings of the conventional methods (see Section Establishment of Structural-functional DMN Template for detail).

Over the past few years, there has been growing interest in using various machine learning methods to analyze the relationship between the cognition and brain networks, possibly providing the mental health assessment model for the psychiatric or potential disorders. For example, support vector machine (SVM) (Vapnik and Chervonenkis, 1991; Cortes and Vapnik, 1995; Vapnik, 2000) has its unique advantages in dealing with the datasets that are small, non-linear, and high-dimensional, and has been widely used in many research directions with the dramatically best results for pattern classification. SVM was originally put forward for supervised two-class training, which required both positive and negative examples as priori knowledge in the training process. For solving the unsupervised problem, Schölkopf et al. (2001) proposed the one-class SVM, which could perform an unsupervised classification with only positive information. Founding on the heuristic of the former researches, in this study, we used the CCs among the DMN regions as the learning features, and established a two-fold SVM-based (TFSVM) classifier model for the mental sub-health active detection, with one-class SVM (OCSVM) utilized to construct the initial classifier and two-class SVM (TCSVM) further used to refine the classification performance. The training process not only made the TFSVM classifier do automatical anomaly detection, but also made it more accurate and robust.

After using the classifier to predict the seafarers' mental health status, we explored the altered DMN activities of the sub-healthy seafarers in contrast to the HC group by using some biomarkers, such as the FC, the regional homogeneity (ReHo), and the fractional amplitude of low-frequency fluctuation (fALFF). The detailed implementation process will be demonstrated later, and finally, results and analysis will be presented together with interpretations and conclusions related to advantages and limitations of this new mental sub-health early warning model.

## Materials and methods

### Experiment data acquisition and preprocessing

#### Seafarers' fMRI dataset

In this research, 79 seafarers [ages between 37 and 57] that participated in the experiment came from a shipping company of Shanghai. They all have 10–20 years sailing experience and came from various positions, such as political commissar, mate, helmsman and seaman, etc. During the data acquisition process, all of the participants were informed about the purpose of this study and given the written informed consent in accordance with the Declaration of Helsinki. The resting-state fMRI data of seafarers were acquired in the Shanghai Key Laboratory of Magnetic Resonance of the East China Normal University. All the participants were instructed to keep the body motionless, eyes closed, relaxed (don't think anything systematically) and awake; their ears were stuffed up with the earplugs in order to reduce effect of the machine noise. BOLD fMRI dataset was acquired using echo planar imaging with 36 slices providing whole-brain coverage and 160 volumes. Other main parameters were listed as follows: GE 3.0 Tesla, gradient echo EPI, *TR* = 2000 ms, matrix size 64×64, in-plane resolution = 3.75 mm × 3.75 mm, and slice thickness = 4 mm.

#### Healthy control group dataset

Seventy four samples [ages between 36 and 58] were selected as the healthy controls in the study. Twenty resting-state fMRI data of them were acquired in the Shanghai Key Laboratory of Magnetic Resonance (SKLMR) of the East China Normal University with the same scan-parameters with the seafarers. The rest were selected from the resting-state fMRI datasets that downloaded from the public neuroimaging database (http://www.nitrc.org/frs/?group_id=296). Table 1 showed the detail information and scan- parameters of those datasets. The healthy controls were used to create the mean normal functional DMN template and to train the two-fold SVM classifier.

**Table 1:** **Information and scan-parameters of healthy control datasets**.

**Dataset**	**Number**	**TR(ms)**	**Volumes**	**Slices**	**Matrix size**	**In-plan resolution(mm × mm)**	**Slice thickness(mm)**
Milwankee_b	39	2000	175	64	36×64	4×3.75	3.75
New_York_a	8	2000	192	39	64×64	3×3	3
New_York_b	7	2000	175	33	64×80	3×3	4
SKLMR	20	2000	160	36	64×64	3.75×3.75	4

#### Data preprocessing

We used DPARSF (http://rfmri.org/DPARSF) for preprocessing. For the seafarer data, the first 10 time points were discarded for scanner calibration and for subjects to get used to the circumstance. For the HC data, the first N time points (*N* = 25, 42, 25, 10 correspond to different dataset) were discarded so that all subjects had the same length of time series in further processing. Then, part of the preprocessing were performed, including slice-timing, head motion correction, nuisance covariates (movement artifacts, white matter and CSF signals) regression, and spatial normalization (resampling voxel size = 2×2×2 mm^3^). Subjects with head motion more than 2 mm or 2° (5 seafarers and 0 healthy controls) of maximal rotation throughout the course of scanning were excluded from further analysis. Then, for FC calculation, the further preprocessing including smooth (FWHM = 5), detrend, and temporal filtering (0.01–0.08 Hz); for ReHo calculation, the further preprocessing including detrend and temporal filtering (0.01–0.08 Hz); for fALFF calculation, the further preprocessing including smooth (FWHM = 5) and detrend.

### Feature extraction

#### Establishment of structural-functional DMN template

According to the existing research results, functional DMN mainly includes the medial prefrontal cortex (MPFC), posterior cingulated cortex (PCC), and bilateral inferior parietal cortex (bIPC). Besides, these brain areas can also be corresponded to prefrontal, parietal, temporal and occipital cortexes in anatomy. The precise positing of DMN is the first critical factor. At present, there are two frequently-used methods for mask construction, one is anatomy-based structural location method by using Anatomical Automatic Labeling (AAL) (Tzourio-Mazoyer et al., 2002) or Brodmann area; the other is functional network detection method base on some data driven method, such as independent component analysis (ICA) (Hyvärinen and Oja, 1997, 2000). However, the DMN structural template defined by AAL contains amounts of regions which may not be detected in the functional activation when using the functional image technology. In addition, the functional template obtained by ICA may include some false positive voxels due to machine or physical noise, and it is difficult to further divide the functional DMN to different brain regions(Buckner et al., 2008). Therefore, in this study, we established the DMN template by combining the functional method with the structural method, which can make up some shortcomings of the conventional methods. The construction process can be described as follows:

- Functional DMN template construction: FastICA package (Hyvärinen and Oja, 1997) was first utilized to process each subject data regarding to the HC group dataset, and after that, we extracted the DMN of each subject. During this procedure, the components' orders of FastICA were automatically estimated by Laplace approximation method (Minka, 2000), which was used in many researches (Wang et al., 2013, 2015a,b). Then the mean DMN was calculated according to all of the extracted DMNs of the healthy control subjects. Meanwhile, the mean DMN was further normalized to the corresponding z-score scale map. Finally, we achieved the functional DMN template by the z-thresh operation with threshold equal to 2.0 (McKeown et al., 1998). In order to facilitate the subsequent template building process, we firstly extracted the voxels with value equal to or greater than 2.0 and then reassigned them to logical value 1.0, whereas the rest set to logical value 0.
- Structural DMN template construction: the four areas including prefrontal, parietal, temporal, and occipital cortices were extracted according to the AAL by utilizing wfu_pickatlas (http://fmri.wfubmc.edu/cms/software) (Maldjian et al., 2003, 2004), with four different areas were marked as 1, 2, 3, 4 respectively.
- Structural-functional DMN template construction: Following the two steps above, we used the “ImaCalc” function of SPM8 (www.fil.ion.ucl.ac.uk/spm/) to construct the structural-functional DMN template by the following steps: first, loading the structural-template and functional-template respectively as mask *i*1 and *i*2; then, calculating the expression *i*1.^*^i2; finally, the structural-functional template was obtained with labels (1, 2, 3, 4) correspond to four different regions.

#### Correlation analysis

Based on the structural-functional DMN template established above, we firstly extracted four ROIs of DMN according to the different labels (1, 2, 3, 4), which represented the prefrontal, parietal, temporal, and occipital cortices respectively. After that, the correlation coefficients of ROIs were calculated as learning features for the training process of TFSVM-based mental health assessment classifier. Here, we used the Pearson's correlation:

(1) $$\begin{array}{rc} \rho_{X,Y}=\frac{cov\left(TC\left(X\right),TC\left(Y\right)\right)}{\sqrt{Var\left(TC\left(X\right)\right)\times Var\left(TC\left(Y\right)\right)}} & \end{array}$$

where *TC*(*X*) and *TC*(*Y*) represent the average time series of ROI_X_ and ROI_Y_; *Var*(*TC*(*X*)) and *Var*(*TC*(*Y*)) represent the variance of the average time course of ROI_X_ and ROI_Y_.

### TFSVM-based mental health assessment

**Figure 1:**
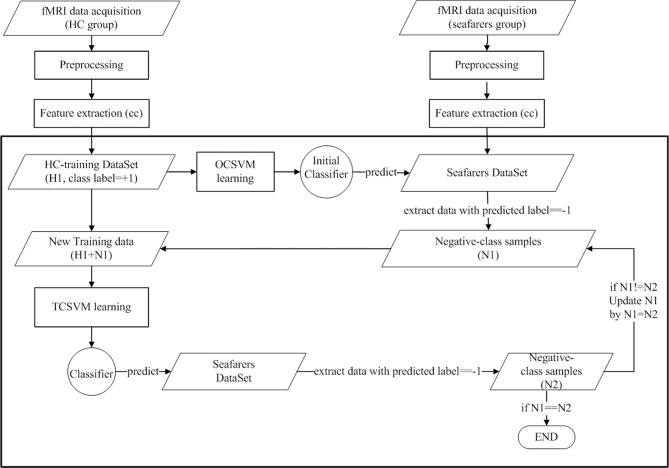
**Implementation diagram of the classification process: OCSVM provides an initial classifier, which is used to predict the class labels of seafarers**. The negative-class samples are selected, together with the healthy control samples whose class labels are set to “1,” as the training data with the class labels are set to “-1.” The TCSVM training process can be repeated to further refine the classifier.

In our study, we assigned the class label “+1” to the HC group, and “−1” to the mental sub-healthy seafarers, which were considered as the negative-class samples. Fifty samples of HC group were randomly selected as the positive-class training set, with the rest as the testing set. Figure 1 illustrated the learning process, and the classifier was obtained mainly by the following steps that showed in the block diagram:

- OCSVM training and classifying process: for the absence of the negative-class samples, the OCSVM was used firstly to construct the initial classifier, with the correlation coefficients among the DMN's areas of the positive-class samples (labeled by “+1”) as the learning features. During this process, grid search (Hsu et al., 2003) with seven-fold cross-validation was utilized to search the best parameters n and g, *n* = 0.01, *g* = 0.009, where *n* represents the *v*∈(0 1) in the OCSVM algorithm, *g* represents the γ in the RBF kernel. The relevant parameters were explained in Appendix—one-class SVM. Then the initial model was used to predict the seafarer-samples, and 5 seafarers were detected as the negative-class samples (mental sub-healthy seafarers), with the class labels set to “−1.” Using the initial OCSVM classifier to predict the 24 testing data, the prediction accuracy was 91.67% with 2 HC were detected as abnormal samples (labeled by “−1”). The FPR (ratio of false positive, which means the normal labeled as the abnormal) of the OCSVM classifier was 8.33%, and the TPR (ratio of true positive, which means the normal labeled as the normal) was 91.67%.
- TCSVM training and classifying process: After the process (i) was implemented, the negative-class samples corresponding to the mental sub-healthy seafarers were retrieved. With combining the negative-class samples (labeled by “−1”) and the 50 HC samples (labeled by “+1”) as new training set, the TCSVM method was utilized in the reclassification process. Furthermore, the TCSVM training and classifying process were repeated to further refine the classification accuracy of the two-fold SVM classifier, until the result was stable, which meant there was no new seafarer sample to be detected as negative. Finally, 10 seafarers were detected as mental sub-health with alterative DMN functional connectivity, and their class labels were set to “−1.” During this process, grid search with five-fold cross-validation was utilized to search the best parameters c and g in every TCSVM training producer. The relevant parameters were explained in Appendix- the introduction of TCSVM algorithm. Using the final classifier to predict the 24 testing data, the prediction accuracy was 95.83% with 1 HC detected as abnormal samples (labeled by “−1”). The FPR of the final classifier was 4.17%, and the TPR was 95.83%.

In a word, the proposed TFSVM classifier fully took advantage of the unsupervised and supervised learning merits of OCSVM and TCSVM respectively, which held more accurate classification performance than the OCSVM did in unsupervised learning and broke through the two class label priori restriction of TCSVM.

### Functional connectivity analysis

Inspired by previous DMN studies which reported that the prefrontal and parietal cortices were the core regions of DMN, we respectively regarded the prefrontal cortex and parietal cortex as seed points and calculated the FC of DMN. The detail procedure could be described as follows: firstly, we respectively extracted the mean time series of the two regions as the seed time series; then, the Pearson correlation coefficient r of the seed time series and every voxel of DMN were calculated; after that, given the value r to the corresponding voxel, and the individual FC maps were obtained; finally, the FC maps were normalized to the corresponding z-score scale maps (z_FC maps) for further two-sample *t*-test analysis.

### ReHo analysis

The ReHo (Zang et al., 2004) was utilized to describe the functional connectivity of regional brain areas, and it was proposed based on the theoretical assumption that a given voxel is temporally similar to those of its neighbors. Kendall's coefficient of concordance (KCC) was used to measure the ReHo of the time series of a given voxel with those of its nearest N neighbors (*N* = 26) in a voxel-wise way.

(2) $$\begin{array}{r} \text{W}=\frac{\sum {\left({\text{R}}_{\text{i}}\right)}^{2}-\text{n}{\left(\bar{R}\right)}^{2}}{\frac{1}{12}{\text{K}}^{2}\left({\text{n}}^{3}-\text{n}\right)} \end{array}$$

where W is the KCC among given voxels, ranged from 0 to 1; R_*i*_ is the sum rank of the *i*th time point; $\bar{R}=\left[\left(n+1\right)K\right]∕2$ is the mean of the *R*_*i*_'s; K is the number of time series within a measured cluster (*K* = 27, one given voxel plus N) and n is the number of ranks (*n* = 150). After that, the KCC value was given to this voxel and individual ReHo maps were obtained. Then the ReHo maps were normalized to z-score maps, and two-sample *t*-test with *p* < 0.05 was performed to explore the altered ReHo of mental sub-healthy seafarers in contrast to HC group. During this procedure, all voxels belonged to the DMN, and the KCC was calculated based on the preprocessed fMRI data without smooth.

### fALFF analysis

The fALFF (Zou et al., 2008) was suggested to reflect the intensity of regional spontaneous brain activity. After the preprocessing, we first extracted voxels that belonged to the DMN, and the time series for each voxel was transformed to a frequency domain without band-pass filtering. After that, the square root was calculated at each frequency of the power spectrum, and the sum of amplitude across 0.01–0.08 Hz was divided by that across the entire frequency range, i.e., 0–0.25 Hz. Then, the ratio was given to this voxel and the correspondingly individual fALFF maps were obtained. Similarly, the fALFF maps were normalized to z-score maps, and two-sample *t*-test with *p* < 0.05 was utilized to explore the altered fALFF of mental sub-healthy seafarers in contrast to HC group.

## Results

After the prediction of the seafarers' mental health status, we evaluated the differences of FC, ReHo and fALFF between the 10 mental sub-healthy seafarers and the HC group by using a two-sample *t*-test with *p* < 0.05. The *t*-test procedures were performed on the individual z-score maps in a voxel-by-voxel manner based on the following formula:

(3) $$\begin{array}{r} T=\frac{{\bar{X}}_{1}-{\bar{X}}_{2}}{\sqrt{\frac{S_{1}^{2}\left(n_{1}-1\right)+S_{2}^{2}\left(n_{2}-1\right)}{n_{1}+n_{2}-2}\left(\frac{1}{n_{1}}+\frac{1}{n_{2}}\right)}} \end{array}$$

where ${\bar{X}}_{1}$ and ${\bar{X}}_{2}$ represented the average of FC, ReHo or fALFF of the mental sub-health seafarers and the HC group respectively; $S_{1}^{2}$ and $S_{2}^{2}$ represented the corresponding variance. Finally, clusters with |*T*| > 2.1 and with a minimum volume of 784 mm^3^ (*P* < 0.05, AlphaSim correction using the REST software, http://www.restfmri.net/forum/rest), were thought to have significant differences between these two groups.

**Figure 2:**
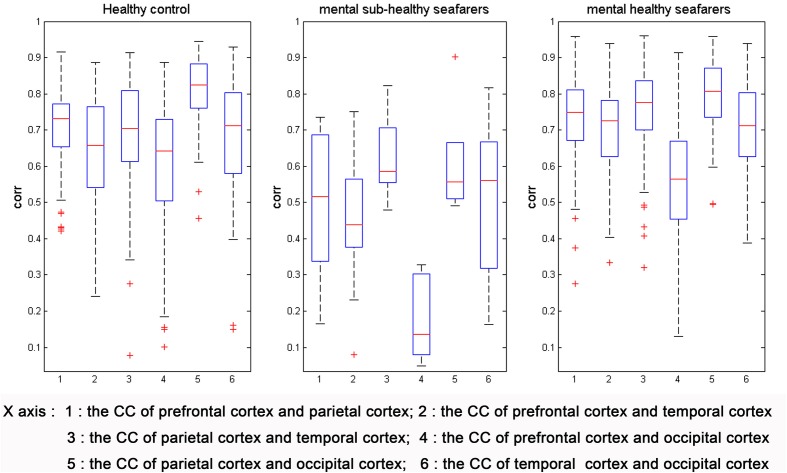
**The distributions of CCs among different DMN regions of the HC group, the mental sub-healthy seafarers, and the mental healthy seafarers**. The CCs of the mental sub-healthy seafarers were significant lower than that of the HC group; the distribution of the CCs of the mental healthy seafarers was similar to that of the HC group.

Figure 2 illustrated the distributions of CCs among different DMN regions of the HC group, the mental sub-healthy seafarers, and the mental healthy seafarers. The number 1–6 along the horizontal axis respectively represented the CC distributions of six pairs of cortices (prefrontal and parietal cortices, prefrontal and temporal cortices, parietal and temporal cortices, prefrontal and occipital cortices, parietal and occipital cortices, temporal and occipital cortices). From this figure, we could observe that the CCs of the mental sub-healthy seafarers were significant lower than that of the HC group; by contrast, the distribution of the CCs of the mental healthy seafarers was similar to that of the HC group.

**Figure 3:**
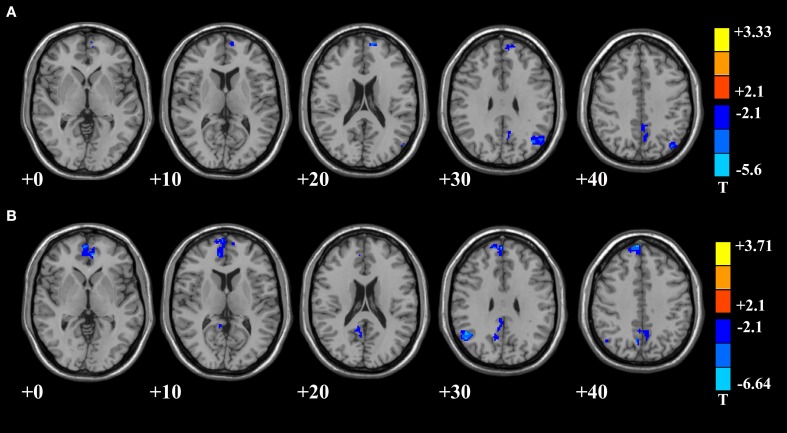
**DMN areas with decreased functional connectivity in mental sub-healthy seafarers. (A)** Regard the prefrontal cortex as seed point with FC decreased in multi-regions of DMN; **(B)** regard the parietal as seed point with FC decreased in the prefrontal and parietal cortices.

The results of two-sample *t*-test on FC maps of the mental sub-healthy seafarers and the HC group were presented in Figure 3, where the prefrontal cortex and the parietal cortex were used as the seed points in (A) and (B) respectively. Compared with the HC group, when taking the prefrontal cortex as seed point, the mental sub-healthy seafarers showed significant decreased FC in multi-regions of the DMN, which were presented in Figure 3A and Table 2 in detail. Similarly, when taking the parietal cortex as seed point, the mental sub-healthy seafarers showed significant decreased FC in the prefrontal and parietal cortices, which were presented in Figure 3B and Table 3 in detail.

**Table 2:** **Prefrontal cortex based DMN areas with decreased functional connectivity in mental sub-healthy seafarers comparing with healthy controls**.

**Anatomical area**	**AAL**	**Number of voxels**	**Peak MNI coordinates(x,y,z)**	**Peak *t* value**
Prefrontal cortex	Frontal_Sup_Medial_L	132	−10, 58, 22	−3.9472
	Cingulum_Ant_L			
	Frontal_Sup_L			
Parietal/ temporal/ occipital cortex	Angular_L Occipital_Mid_L Temporal_Mid_L Parietal_Inf_L	345	−52, −68, 26	−5.6042
Parietal cortex	Precuneus_L/R Cuneus_L Cingulum_Post_L	226	−4, −56, 46	−4.4093

*R, right; L, left; AAL, Anatomical Automatic Labeling atlas; MNI, Montreal Neurological Institute*.

**Table 3:** **Parietal cortex based DMN areas with decreased functional connectivity in mental sub-healthy seafarers comparing with healthy controls**.

**Anatomical area**	**AAL**	**Number of voxels**	**Peak MNI coordinates(x,y,z)**	**Peak *t*-value**
Prefrontal cortex	Frontal_Sup_Medial_L/R	451	−6, 46, 0	−5.2889
	Cingulum_Ant_L/R			
	Frontal_Sup_L/R Frontal_Med_Orb_R			
Parietal cortex	Precuneus_L/R Cuneus_R	183	8, −66, 40	−4.7463
Parietal cortex	Angular_R	220	50, −58, 30	−6.6391
Parietal cortex	Cingulum_Post_L/R	179	−4, −56, 44	−3.4711
	Precuneus_L/R			
	Cingulum_Mid_R			
Prefrontal	Frontal_Sup_Medial_L/R Frontal_Sup_R	366	8, 58, 38	−6.3596

*R, right; L, left; AAL, Anatomical Automatic Labeling atlas; MNI, Montreal Neurological Institute*.

**Figure 4:**
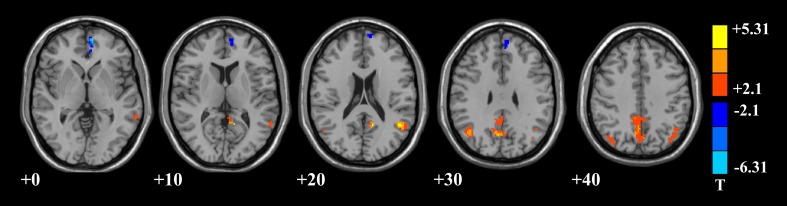
**DMN areas with decreased and increased regional homogeneity in multi-regions of DMN regarding to the mental sub-healthy seafarers**.

**Figure 5:**
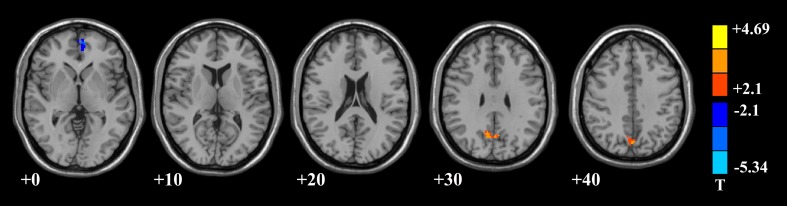
**DMN areas with decreased fractional amplitude of low-frequency fluctuation (fALFF) in the prefrontal cortex, and increased fALFF in the parietal cortex regarding to the mental sub-healthy seafarers**.

In addition, we further calculated the local features, such as ReHo and fALFF, of DMN to investigate the differences of brain functional activities between the mental sub-healthy seafarers and the HC group. The mental sub-healthy seafarers showed a decreased ReHo in parts of prefrontal cortex and increased ReHo in many sub-regions of the parietal, temporal and occipital cortices (Figure 4, Table 4). In addition, the mental sub-healthy seafarers also showed a decreased fALFF in some areas of the prefrontal cortex and increased fALFF in parts of the parietal cortex (Figure 5, Table 5).

**Table 4:** **DMN areas with decreased or increased regional homogeneity in mental sub-healthy seafarers comparing with healthy controls**.

**Anatomical area**	**AAL**	**Number of voxels**	**Peak MNI coordinates(x,y,z)**	**Peak *t*-value**
Prefrontal cortex	Frontal_Sup_Medial_L Cingulum_Ant_L Frontal_Sup_L	239	−8, 54, 2	−6.3117
Parietal/temporal cortex	Temporal_Mid_L Angular_L	235	−48, −54, 22	4.9141
parietal cortex	Precuneus_L/R	922	−12, −54, 18	5.309
	Cingulum_Post_L/RCingulum_Mid_RCuneus_L/R			
parietal/temporal cortex	Angular_R Occipital_Mid_R Parietal_Sup_R Temporal_Mid_R Occipital_Sup_R	304	36, −74, 50	4.2039
parietal/occipital cortex	Angular_L Parietal_Inf_L Occipital_Mid_L	141	−46, −60, 42	3.3074

*R, right; L, left; AAL, Anatomical Automatic Labeling atlas; MNI, Montreal Neurological Institute*.

**Table 5:** **DMN areas with decreased or increased fractional amplitude of low-frequency fluctuation (fALFF) in negative-class seafarers comparing with healthy controls**.

**Anatomical area**	**AAL**	**Number of voxels**	**Peak MNI coordinates(x,y,z)**	**Peak *t*-value**
Prefrontal cortex	Frontal_Sup_Medial_L/R Cingulum_Ant_L/R	120	−4, 58, 2	−4.2828
Parietal cortex	Precuneus_L/R Cuneus_L/R	217	0, −68, 28	4.4146

*R, right; L, left; AAL, Anatomical Automatic Labeling atlas; MNI, Montreal Neurological Institute*.

## Discussion

Mental sub-health is detrimental not only to the sufferers, but also to their families, their friends, and even the society. Early warning for mental disorders is very important, promising and challenging. Based on the previous researches, the resting-state cognition was shown to correlate with mental health (Diaz et al., 2013), and the DMN activity has been proven to be closely associated with human higher cognition (Buckner et al., 2008), such as many potential functions, ranging from internal processes, e.g., self-reflection, to diffused passive attention. Abnormal DMN functional activities could reveal some psychiatric or psychoanalytic disorders, such as the Autism (Wass, 2011), Mild cognitive impairment (Rombouts et al., 2005), Major depression (Greicius et al., 2007), Bipolar disorder (Strakowski et al., 2004), Anxiety (Etkin and Wager, 2007) and Parkinson disease (van Eimeren et al., 2009), etc. Thus, our study took the disordered functional connectivity of DMN as the criterion for abnormal DMN functional activities, which may result in mental sub-health status. Here, the FCs were expressed as the CCs among different DMN regions, which were further utilized as the learning features in the training process of mental health assessment classifier by using the proposed TFSVM method. During this training procedure, for the absence of adequate prior knowledge, OCSVM was firstly utilized for the training of the initial classifier (with the predicted accuracy of 91.67% using in the testing dataset), which predicted 5 seafarers as negative class samples. However, the OCSVM was primitively presented for outlier detection. According to its optimization problem (see Appendix—one-class SVM), the parameter v controlled the upper bound of the classification error ratio and the lower bound of the numbers of the support vectors. Thus, even though all of the training samples were labeled by “+1,” there were always some subjects, who were detected as outliers (labeled by “−1”) via OCSVM due to that v could not be zero. Besides, the results of the OCSVM classification were sensitive to the given *v*-value, and might contain some error messages. Therefore, after the OCSVM training procedure, the TCSVM was further utilized to refine the classifier's performance. The final classifier's prediction accuracy for the testing dataset increased to 95.83%, and 10 seafarers were lastly labeled as negative class samples.

After the assessment process, we evaluated the differences of DMN between the 10 mental sub-healthy seafarers and the HC group. The result showed that when compared with the HC group, the CCs among the different DMN regions regarding to the mental sub-healthy seafarers were significant lower. However, there were no significant differences between the mental healthy seafarers and the HC group. In addition, we also evaluated the FC, ReHo and fALFF differences between the two groups by using a two-sample *t*-test. The *t*-test results showed that when comparing with the HC group, there were significant disorders of DMN functional activities for the mental sub-healthy seafarers. When taking the mean time series of the prefrontal cortex as the seed time series, there was significant decreased FC in multi-regions of the prefrontal, parietal, temporal and occipital cortices. When taking the mean time series of the parietal cortex as the seed time series, there was significant decreased FC in multi-regions of the prefrontal and parietal cortices. Besides, the mental sub-healthy seafarers also showed decreased ReHo and fALFF in parts of the prefrontal cortex, increased ReHo in many other regions of the DMN, and decreased fALFF in sub-regions of the parietal cortex. Those regions of DMN where the disorders occurred in were relevant to the higher cognitive activities of the human beings, such as the memory, the self-examination, monitoring the external environment, and the ability of logical thinking, etc., which might reflect in the mental health status. As Diaz et al. (2013) reported, the Comfort clearly focused on physical and mental well-being, and we speculated that the functional activities of DMN were stable and normal with the Comfort phenotype. Thus, the disordered phenomenon could reflect the mental sub-healthy or unhealthy status. In order to further verify the prediction results, we analyzed the assessment results regarding to the SCL-90 (Symptom Checklist 90) testing for the mental sub-healthy seafarers, and the results showed that most of these mental sub-healthy seafarers had a mild form of some mental dysfunctions, such as obsession, depression, anxiety, hostile or bigotry, etc., which validated that the proposed TFSVM-based classifier could be effectively used to the anomaly detection and was of great significance to the early warning for human mental sub-health.

Based on the fact that our method has showed a good performance at outlier detection, for perfecting the mental sub-health early warning mechanism, there still other works should be done in the next study process. Firstly, considering that some of the fMRI-data acquisition parameters may affect the analysis results, we will collect more HC data and seafarers data with the same age range and scanning parameters to justify and optimize the classifier's performance in the future study. Secondly, according to Diaz and his colleagues report, the resting-state cognition can be characterized by seven phenotypes (Diaz et al., 2013). For guaranteeing the rigor of the study's result, we will take the results of the Amsterdam Resting-State Questionnaire (Diaz et al., 2013) regarding to the HC and the testees as reference factors. Thirdly, apart from the FC of DMN, the alterative activities of other brain areas and the information interaction between DMN and other brain areas also need to be concerned; and how those disordered brain function activities reflect in human's mental state will be further tracked and explored.

In conclusion, the results of our experiment proved that using fMRI technology and two-fold SVM model would be promising to establish a quantitative early warning model for human mental sub-health status. Such a learning method is objective and effective for assessing people's mental health, and is of profound significance for psychology research.

### Conflict of interest statement

The authors declare that the research was conducted in the absence of any commercial or financial relationships that could be construed as a potential conflict of interest.

## Appendix

### Support vector machines

#### Two-class SVM

In machine learning, the TCSVM is a supervised learning method and widely used in pattern recognition field. For a training set {(*x_i_*, *y_i_*), *i* = 1, …, *n*}, $x_{i}\in R^{N}$, *y*_*i*_ ∈ {-1, 1}, where *x*_*i*_ represents a feature vector with *N* dimensions and *y*_*i*_ represents its class label. The TCSVM method aims to find a linear classification hyperplane that maximizes separation of the two classes, which means to maximize the margin magnitude 2∕∥*w*∥ subject to *y*_*i*_((*x*_*i*_ · *w*) + *b*) ≥ 1, where *w* and *b* determine the hyperplane *w* · *x* + *b* = 0. For improving generalization performance or classify non-separable samples, training error is permitted by introducing a slack variables ξ_*i*_ ≥ 0. We can obtain the hyperplane by the calculation as follows:

(1) $$\begin{array}{r} \text{min }C\overset{N}{\underset{i=1}{\sum }}\xi_{i}+\frac{1}{2}∥w{∥}^{2}, \end{array}$$

(2) $$\begin{array}{r} \text{subject to }y_{i}\left(\left(x_{i}\cdot w\right)+b\right)\geq 1-\xi_{i}, \end{array}$$

where *C* is called penalty factor, and it controls the tradeoff between the training error and hyperplane complexity, with a small *C* value corresponding to a large margin size. By solving the Lagrangian dual of (1) and (2), the final decision function can be written as:

(3) $$\begin{array}{r} f\left(x\right)=sgn\left(\overset{N}{\underset{i=1}{\sum }}\alpha_{i}y_{i}\left(x\cdot x_{i}\right)+b\right), \end{array}$$

where α_*i*_ is Lagrange multiplier, (·) is the inner product, and $w=\overset{N}{\underset{i=1}{\sum }}\alpha_{i}y_{i}x_{i}$. The linear SVM can be extended to the non-linear case by using kernel methods which can project the original data into a higher dimensional feature space, and the decision function can be represented as:

(4) $$\begin{array}{r} f\left(x\right)=sgn\left(\overset{N}{\underset{i=1}{\sum }}\alpha_{i}y_{i}k\left(x,x_{i}\right)+b\right), \end{array}$$

where *k*(*x*,*x*_*i*_) = (φ(*x*)·φ(*x*_*i*_)) is a kernel function, and in our study we used the Radial Basis Function (RBF) kernel which defined as $k\left(x,x_{i}\right)=e^{-\gamma ∥x-x_{i}{∥}^{2}}$, where γ is the width parameter, and a large γ value corresponds to a small kernel width.

### One-class SVM

Different with the basic SVM algorithm, the training data of one-class SVM only come from one set with same label. We consider training data *x*_1_,*x*_2_, ⋯  , *x*_*n*_ ∈ *X*, where *n*∈*N* is the number of observations, and *X* ∈ *R*^*N*^ is some set. OCSVM returns a function *f* that takes the value “+1” in a “small” region capturing most of the data points, and “−1” elsewhere. Here we also use the RBF kernel to map the data into a higher dimensional feature space. We can obtain the hyperplane by the calculation as follows:

(5) $$\begin{array}{r} \text{min }\frac{1}{2}∥w{∥}^{2}+\frac{1}{vn}\overset{N}{\underset{i=1}{\sum }}\xi_{i}-\rho , \end{array}$$

(6) $$\begin{array}{r} \text{subject to }\left(w\cdot \varphi \left(x_{i}\right)\right)\geq \rho -\xi_{i},\xi_{i}\geq 0, \end{array}$$

Since non-zero slack variables ξ_*i*_ is penalized in the objective function, we can expect that if *w* and ρ solve the problem, then the decision function

(7) $$\begin{array}{r} f\left(x\right)=sgn\left(w\cdot \varphi \left(x\right)-\rho \right), \end{array}$$

will be positive for most examples *x*_*i*_ contained in the training set, while the ∥*w*∥ will still be small, and *v* controls the upper bound of the classification error ratio and the lower bound of the numbers of the support vectors.

By solving the Lagrangian dual of (5) and (6), and together with the RBF kernel, the final decision function can be written as:

(8) $$\begin{array}{r} f\left(x\right)=sgn\left(\overset{N}{\underset{i=1}{\sum }}\alpha_{i}k\left(x,x_{i}\right)-\rho \right), \end{array}$$

where α_*i*_ is Lagrange multiplier, and *k*(*x*,*x*_*i*_) = (φ(*x*_*i*_)·φ(*x*)).
